# Viscosupplementation in the upper and lower compartments of the temporomandibular joint checked by ultrasonography in an ex vivo and in vivo study

**DOI:** 10.1038/s41598-022-21781-5

**Published:** 2022-10-26

**Authors:** Eduardo Januzzi, Thays Crosara Abrahão Cunha, Graziella Silva, Beatriz Dulcinéia Mendes Souza, Adriana Soares Bicalho Duarte, Marcella Rezende Serpa Zanini, Adriana Maria Andrade, Alexsander Ribeiro Pedrosa, Antônio Luís Neto Custódio, Maurício Augusto Aquino Castro

**Affiliations:** 1 Orofacial Pain Center of the Mater Dei Hospital, Belo Horizonte, Minas Gerais Brazil; 2 Federal University of Uberlândia, Uberlândia, Minas Gerais Brazil; 3 European Centre of Dentistry Speciality, Lisbon, Portugal; 4 Federal University of Santa Catarina, Florianópolis, Santa Catarina Brazil; 5 Faculty of Sete Lagoas, Sete Lagoas, Minas Gerais Brazil; 6 Private Clinic Radiscan, Belo Horizonte, Minas Gerais Brazil; 7 Mater Dei Hospital, Belo Horizonte, Minas Gerais Brazil; 8 Biological Sciences Institute of the Federal University of Minas Gerais, Belo Horizonte, Minas Gerais Brazil; 9 Department of Dental Clinics, Oral Pathology and Oral Surgery of the Faculty of Dentistry of the Federal University of Minas Gerais, Av. Antônio Carlos, 6627, Pampulha, Belo Horizonte, Minas Gerais CEP: 31.270-901 Brazil

**Keywords:** Physiology, Anatomy, Health care, Rheumatology, Signs and symptoms

## Abstract

Viscosupplementation (VS) of the temporomandibular joint (TMJ) aims to treat temporomandibular dysfunction (TMD) by stimulating synovial cells to improve intracapsular lubrication. The purpose of the present study was to assess a VS protocol planned with the aid of cone-beam computed tomography (CBCT) and checked by ultrasonography (US). The study was carried out in 3 stages. The first was to check the correspondence between the proposed facial reference points and the osseous components of the joint by means of CBCT. In the second stage, the upper and lower compartments of 20 TMJs of fresh frozen cadavers were injected with coloured liquids, and the accuracy of the technique was confirmed by dissecting the anatomical specimens. The third stage consisted of VS in 10 patients (20 TMJs), with real-time verification of the location of the needle tip by means of ultrasonography. CBCT confirmed the correct locations of the marked points used in the proposed VS protocol. The dissections showed that 13 of the 14 injections effectively reached the upper and lower compartments. The location of the needle tip was effectively verified by ultrasonography, confirming the correct access to both compartments. The proposed protocol was effective for accessing the upper and lower compartments of the TMJ. The evaluated protocol proved to be accurate, safe and clinically reproducible means of VS in the upper and lower compartments of the TMJ.

## Introduction

Temporomandibular dysfunction (TMD) is characterized by a diverse group of alterations that affect the temporomandibular joint (TMJ), masticatory muscles and associated structures^1,2^. TMD is divided into two main categories, namely, muscular and joint disorders, each of which can be classified into further subdivisions^3–5^. Intracapsular alterations can be related to changes in the synovial liquid^6^.

TMJ viscosupplementation (VS) is a minimally invasive procedure that consists of an intra-articular injection of sodium hyaluronate (HA) into the joint compartments. This treatment has been applied as an efficient therapy to promote articular homeostasis and analgesia by stimulating type B synoviocytes to produce additional synovial liquid, which results in improvements on the rheological properties of the synovial liquid, superior lubrication, absorption of functional loads, and stimulation of chondrocyte proliferation in fibrocartilage^6–11^.

In the application of VS to the TMJ, the best results in terms of pain reduction, increased maximum mouth opening and structural gains are achieved when both the upper and lower compartment are treated^6–12^. However, access the lower compartment is a very difficult procedure owing to the small size of this compartment and the lack of an effective and reproducible method^8^.

VS can be performed based on the identification of anatomical references, or ultrasound guidance^13^. The present study aimed to evaluate a new method for accessing the upper and lower compartments of the TMJ, with validation of the access points by means of cone beam computed tomography (CBCT), since this imaging technique is a reliable means of visualizing the osseous components of the TMJ^14^.

The procedures were also tested in fresh frozen cadavers and later performed in vivo. During the in vivo procedure, each step was verified by goal-oriented ultrasonography (US), which was was selected for its safety and real-time imaging capabilities. This procedure was designed to be suitable for clinical application in dentists’ offices, as the reference points for entrance into the upper and lower joint compartments can be easily and safely identified, and the needle can be guided by US, a rapid and low-cost imaging modality that does not use ionizing radiation^15,16^.

## Results

**Figure 1:**
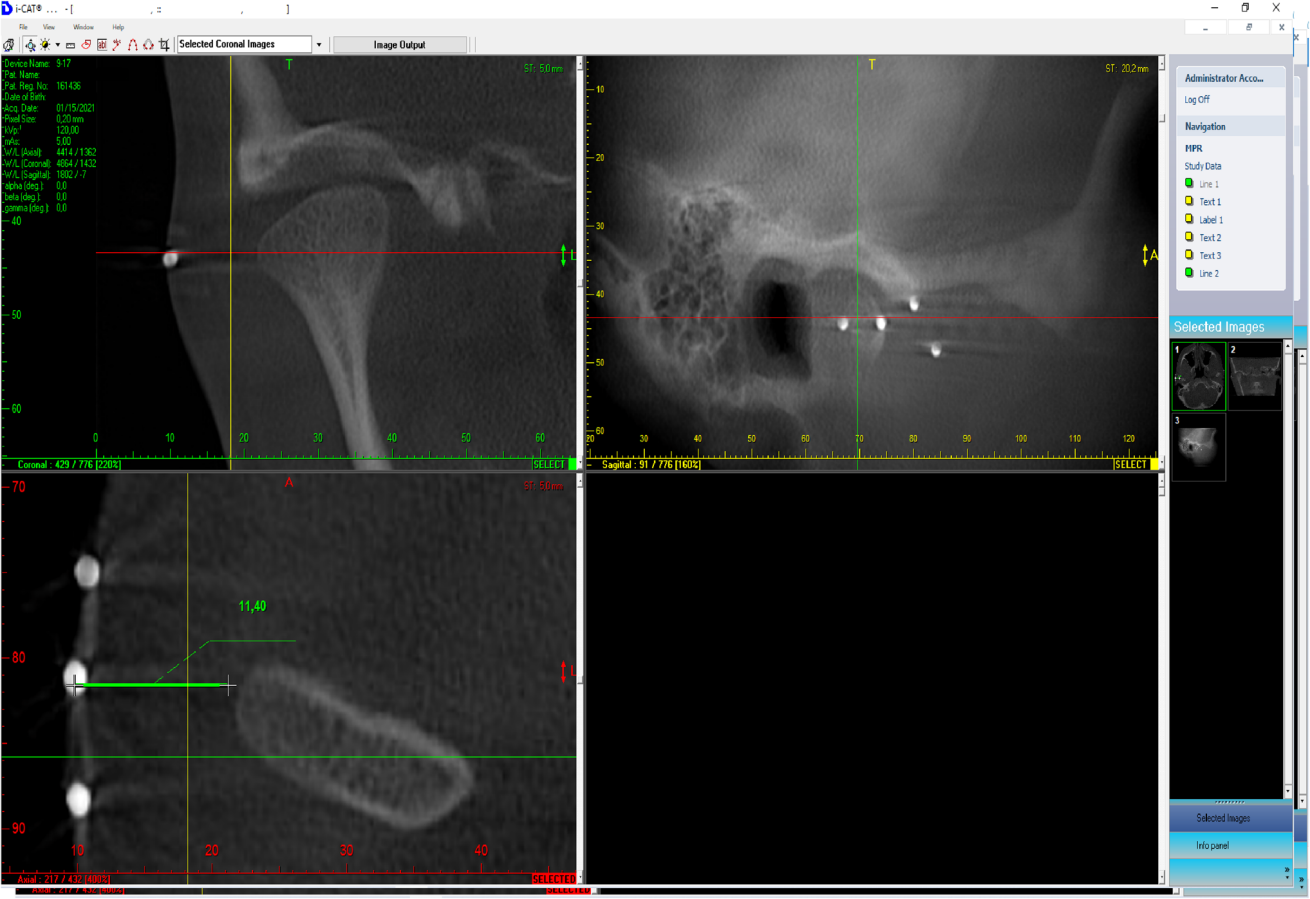
CBCT image*s* showing the correspondence *between* the marked points and *the* osseous joint components.

CBCT scans that the points marked on the surface of the skin were correctly placed with respect to lateral pole of the mandibular condyles in the second stage of the ex vivo study (Fig. 1).

**Figure 2:**
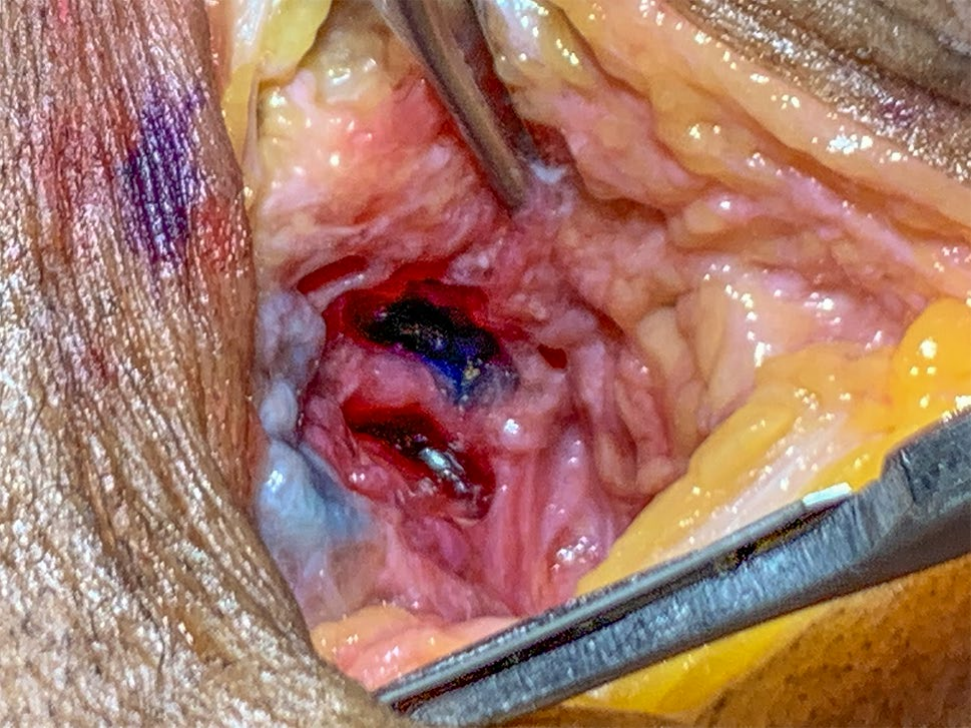
Coloured fluids located in the upper and lower compartments of the TMJ.

In the same stage of the pilot study, the anatomical dissections performed after injections of the remaining 14 TMJs showed that the red and blue fluids were correctly injected into 13 of the 14 joints (92.85% effectiveness) (Fig. 2).

**Figure 3:**
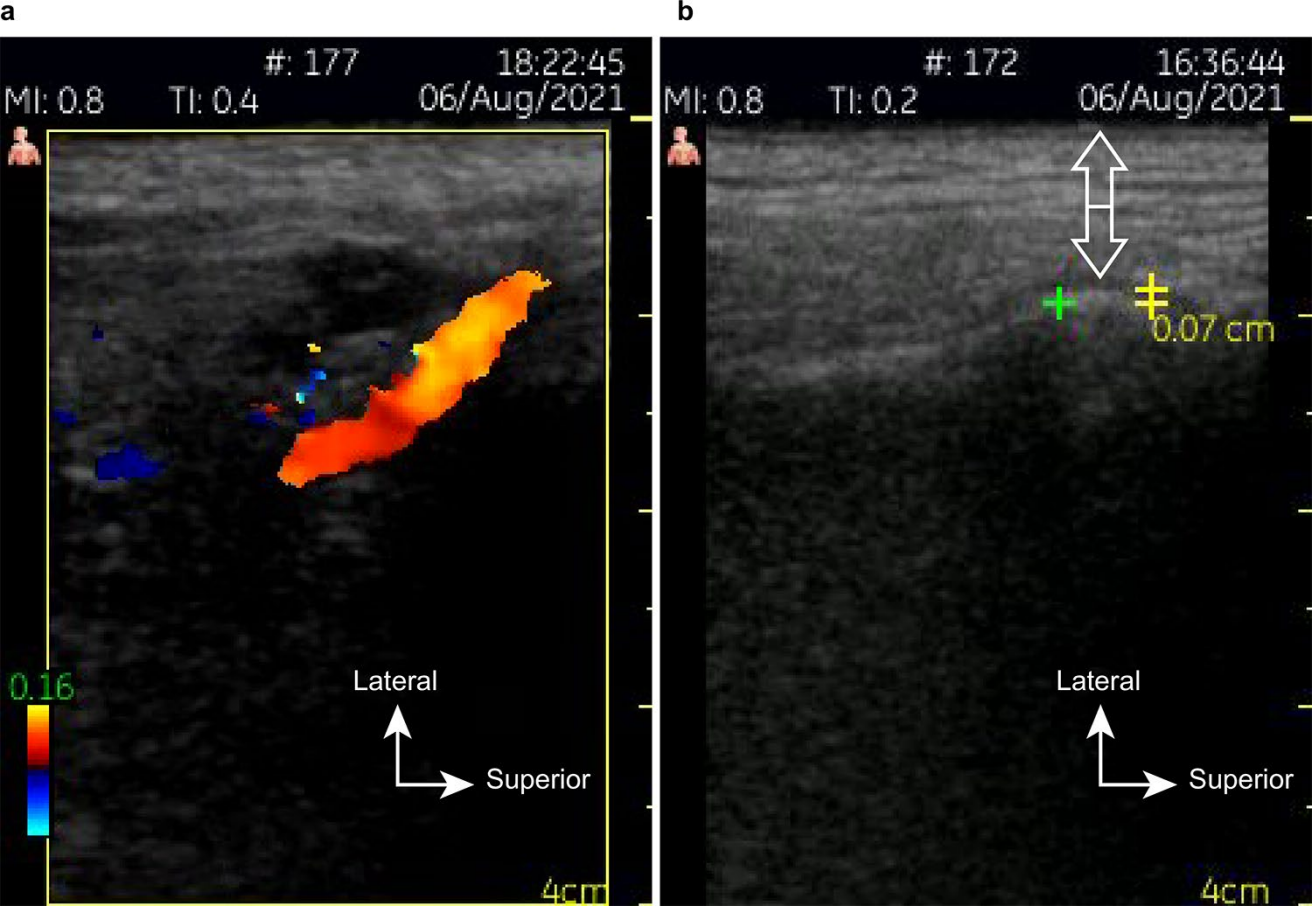
US images showing (**a**) the superficial temporal artery and (**b**) the soft tissue measurements (white arrows) and lateral joint spaces (yellow).

The US evaluation prior to the clinical procedure was able to measure the thickness of the skin and subcutaneous tissues to be punctured, as well as map vascular structures, such as the superficial temporal artery and the transverse facial artery (Fig. 3a,b). These measurements were useful for selecting the appropriate size of the needle and avoiding intravascular infiltration.

Likewise, the US scans were able to check the correct position of the of the needle tips on the lateral surface of the mandibular condyles at the opening of the joint compartments.

**Figure 4:**
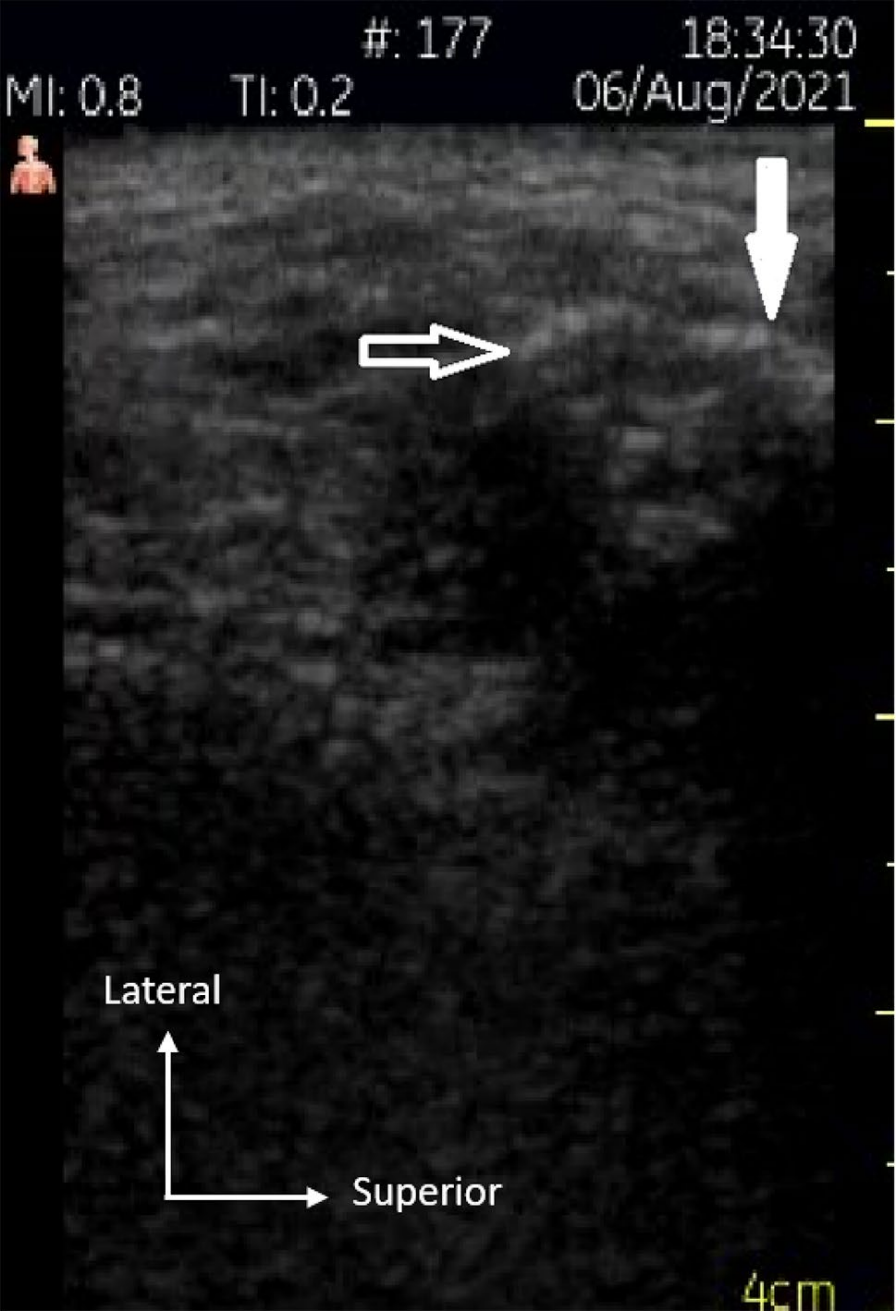
Hyaluronic acid filling the lower compartment over the articular surface (white arrow) and lateral infundibulum (outlined arrow).

During the procedure, it was possible to visualizae the HA filling the space along the superolateral surface of the mandibular condyle, in the same format of the lower compartment of the TMJ. Dynamic real-time images were captured (Video 1, Fig. 4).

**Figure 5:**
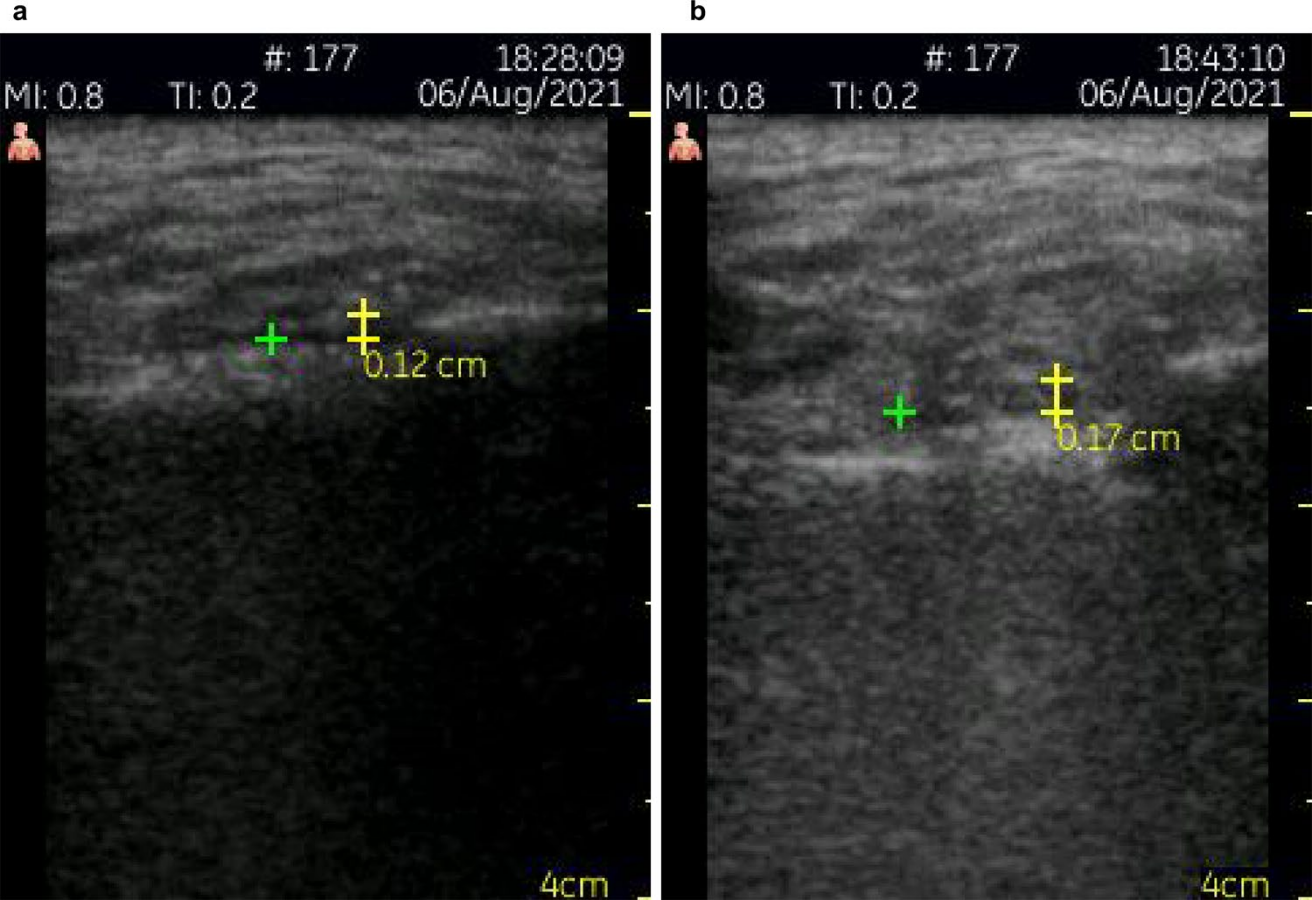
Preoperative (**a**) and postoperative (**b**) measurements of the thickness of the lateral joint space.

A comparison of the preoperative and postoperative measurements showed an increase in the thickness of the lateral joint spaces (Fig. 5). The mean difference between pre and postoperative US measurements was 0.05 mm.

## Discussion

Many studies have demonstrated the benefit of VS in patients with TMDs^6–9,17,18^. Given the importance of VS as a therapeutic resource, it would be advantageous to adopt an easy, predictable, and reproducible protocol so that the procedure can provide the intended benefits^7–9,19^. However, there is still no standardized method that would ensure correct access to upper and lower articular compartments, as with some other well-established infiltration protocols for other joints of the human body.

Accurately identifying the access points for upper and lower compartments of the TMJ contributes to the improvement of this technique and its clinical results. VS presents some potential risks of complications, such as strokes or damage to the joint capsule and collateral articular disk attachments, especially when successive infiltration attempts are conducted. In addition to being ineffective, extracapsular injections can also lead to undesired complications and unsatisfactory effects^20^.

In the evaluation of the proposed protocol, we took advantage of the diagnostic accuracy of CBCT to verify the spatial relationships between the marked points and the osseous joint structures^14^. This ex vivo procedure validated the accuracy of the proposed protocol and its basic premises of making the VS procedure clinically safe and easy. This verification contributed to the achieved results, mainly by confirming reliable, standardized and reproducible entrance points for accessing the joint compartments, especially the lower compartment, which was accessed through the point located 7 mm below Point 2. No difficulty was reported in applying the technique during the injections. Since CBCT is usually requested to complement the clinical evaluation of TMD, the same tomographic examination can be used to verify the marked points in a real situation of clinical care, avoiding additional exposure merely to confirm the correct placement of the points^8,10,18,21^. Magnetic resonance imaging (MRI) could provide a better evaluation of the marked points in relation to upper and lower compartments, since it provides a better view of the articular disc and its attachments^2,6^. However, the use of metallic microspheres and the unavailability of the equipment for evaluating heads of fresh cadavers hindered the use of MRI in the present study. The authors suggest that this evaluation should be conducted in future studies, despite its higher financial cost.

The anatomical dissections of the TMJs performed during the ex vivo phase made it possible to validate the accuracy of the proposed protocol in an unambiguous manner. Colored fluid was used due to its adequate flow and consistency for injection, which allowed dissections without displacement or loss^13^. From The colours, contrasting with the surrounding tissues, facilitated visual identification and demonstrated the protocol’s effectiveness in targetting the upper and the lower compartments separately. The dissections indicated that point 4, located 7 mm below Point 2 provided highly reliable access to the lower compartment.

Regarding preoperative US examination, this step made it possible to measure the thickness of the soft tissues to be punctured, helping the surgeon select the correct needle length and avoid extra-articular infiltration of HA, as well as possible injuries of the intracapsular components caused by introducing an excessively long needle. The variation of the pressure between the transducer and the skin, and the colour Doppler mode applied in the preoperative evaluation enabled the identification of blood vessels in the region, which could represent a potential risk of intravascular HA injection, haemorrhage or haematomas. The presence of anatomic variability, related to variable positioning of the TMJ in relation to individual facial phenotypes, made prior US evaluation a highly useful procedure for the execution of safe and truly minimally invasive VS, in accordance with point-of-care US recommendations^15,16,22^. Injections in the upper compartment using Point 1 proved to be relatively simple. The direct access to this compartment when the mouth is open allows the easy, safe and effective execution of this procedure. The anterior articular disc displacement and the distension of the posterior attachments facilitate the puncture^13^.

The smaller dimensions of the lower compartment, by contrast, create considerable difficulty for precise infiltration^8^. Although the dissections have demonstrated that the lower puncture point was appropriate, errors in positioning the needle’s tip can occur. In this sense, real-time US verification promoted greater safety to both operator and patient. With the stable positioning of the open mouth, promoted by a 10 mm interincisal spacer, a careful manoeuvre to guide the needle tip in the posterior direction, close to the mandibular condyle posterior profile, followed by a discrete medial deepening, into the most inner portion of the lower compartment, until a tactile perception of penetration occurs, results in a safe procedure, with excellent predictability and low morbidity.

Although the US images are not capable of showing the upper and lower compartments of the TMJ in their entirety, as highlighted by Friedman et al.^23^, the increase in the lateral joint space between pre- and postoperative measurements allowed us to infer the success of infiltration. An increase in the lateral space thickness in the postoperative examination indicates the precise injection of HA.

The reports of immediate relief of the painful symptoms after the procedures, in addition to an increase in maximum mouth opening, also indicate that the therapy is effective and can be considered adequate, safe, and predictable with low morbidity. The signs and symptoms of displacement of the disc are reduced after VS, which confirms the possibility of effectiveness in the biomechanical recovery of the joint as well as in degenerative alterations^6^. The beneficial effects of the viscosupplement, such as the reduction in intracapsular friction, improvement in the rheological environment, and induction of the production of endogenous HA, contribute to the anatomic and functional (biomechanical) rearrangement. Future studies must be performed to prove these observations.

In relation to US video (Video 1), showing the viscosupplement being injected, filling the upper and lateral portions of the acoustic shadow of the mandibular condyle, its aspect corresponds to a shape morphologically compatible with the lower compartment, filling the space above the articular surface of the mandibular condyle and laterally to the mandibular condyle (Fig. 4). This finding is in accordance with reports by Alomar et al.^24^ and by Willard et al.^25^, who verified that the insertion of the lateral disc attachments occurs at its edge and extends below the lateral pole of the mandibular condyle, causing the occurrence of a peculiar extension of the lower compartment, bypassing the lateral pole of the mandibular condyle.

**Figure 6:**
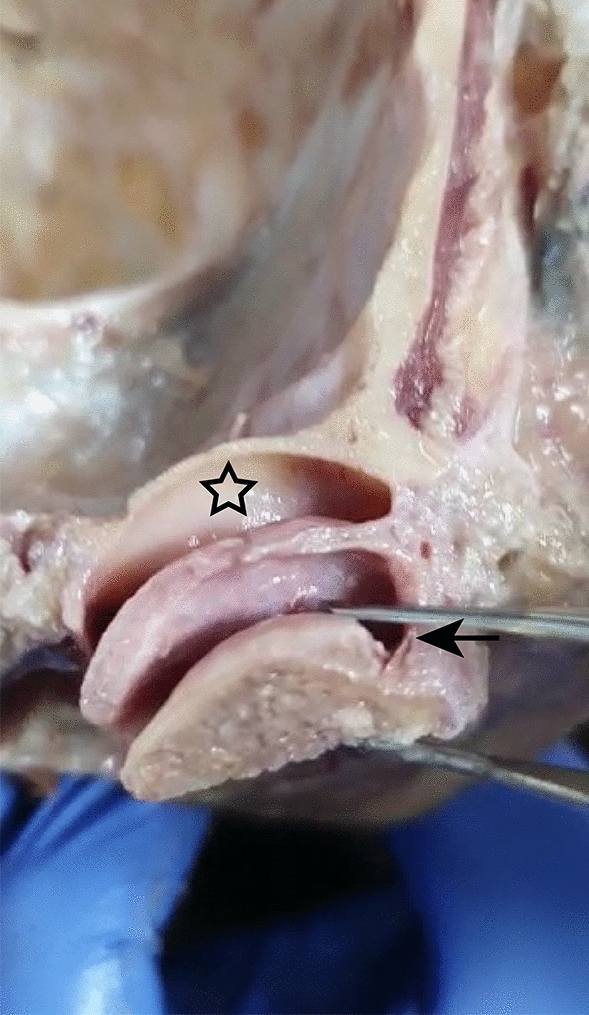
Dissection of a fresh frozen cadaver TMJ showing the upper compartment (asterisk) and the lateral infundibulum of the lower compartment (outlined arrow).

This anatomical finding has not yet been reported in the literature and has been designated by the present authors as the lateral infundibulum of the lower compartment. Its morphology is identical to that presented by the viscosupplement in the obtained ultrasound video. To confirm the existence of this lateral infundibulum, TMJ dissection of a fresh cadaver was performed, clearly showing the same shape found in the US video and confirming the correct insertion of the HA in the lower compartment (Fig. 6). This image provides unequivocal verification of the precise infiltration in the lower compartment, as it differs morphologically from the aspect of the upper compartment.

The explanation for viewing a hyperechoic image of the HA may be related to a local factor or an isolated cause. Microbubbles may have been generated due to the negative pressure in the inner portion of the syringe, conducted during the withdrawal of the plunger for suction prior to the injection. This procedure, performed in order to verify the correct intracapsular positioning of the needle tip may have caused the emergence of microbubbles, which readilly reflect ultrasound waves. It is not possible to predict if the same effect can occur with different concentrations of HA. Studies on the effect of exogenous HA with different molecular weights suggest that the high molecular weight is important in the lubrication and protection of intracapsular structures^6,26–30^.

Clinical procedure and do not observe some principles of minimally invasive procedures.

## Conclusion

In conclusion, the evaluated protocol was effective for accessing the upper and lower compartments of the TMJ. The CBCT and US evaluation, as well as the dissections, contributed to verifying its accuracy. The procedures described here can be easily, safely, and predictably performed based on the clinical identification of well-established anatomical references, which reduces the necessary learning time.

As this method does not present known risks to human health and is widely available, the authors hope that it will be widely implemented. However, it is necessary to consider the learning time required to handle a US device and interpret the images correctly.

## Methods

Ethical approval for the study was obtained from the ethical committee of the Federal University of Minas Gerais (CAAE: 53014921.5.0000.5149). All methods were performed in accordance with the relevant guidelines and regulations.

Based on the methods previously proposed by Cha et al. ^13^ and Honda and Bjørnland ^21^ a new protocol was proposed to access the upper and lower compartments of the TMJ. This protocol consists of the following steps:

1. Trace a straight line on the skin from the tragus to the lateral contour of the orbit (Tr-OL line), with the aid of a flexible plastic ruler and a washable ink pen.
2. With the patient’s mouth open, identify the pre-auricular depression, formed in the soft tissues by the anterior translation of the mandibular condyle. Mark the point on the Tr-Ol line where it crosses the pre-auricular depression (Point 1); this location serve as the access point to the upper compartment of the TMJ when the mouth is open.
3. Mark two additional points on the Tr-Ol line, 5 mm and 10 mm anterior to Point 1 (Points 2 and 3).
4. Position the ruler on point 2, perpendicular to the Tr-Ol line, and mark a point located 7 mm below (Point 4).
5. Position the ruler perpendicular to the Tr-Ol line, intersecting with it at Point 3, and mark a point located 7 mm below (Point 5).
6. Inject 0.3 ml of local anesthesia without vasoconstrictors in the subcutaneous tissues underlying Point 4 or 5 (0.3 ml). After the anaesthetic takes effect, stabilize the mouth in an open position with the aid of a 10 mm interincisal bite block to promote anterior translation of the mandibular condyle and expansion of the lower intracapsular space.
7. To access the lower compartment, insert a needle (0.55 × 20 mm–24G × 3/4″), at Point 4. The needle should be positioned with the bevel facing the patient’s skin, and inserted with a 45-degrees slope in the inferior-superior direction (caudal–cranial). Resistance corresponding to a slight touch on the lateral surface of the mandibular condyle will help confirm the correct placement of the needle tip. If this resistance does not occur, remove de needle and repeat the procedure at Point 5. The point with the greater bone area and stability should be selected for VS in the lower compartment. When the needle touches the lateral surface of the mandibular condyle, check the correct positioning of the needle tip at the entrance to the lower compartment by means of real-time US imaging. Adjust the position of the needle tip when necessary. Once the needle location is correct, pull the plunger, which should return to its initial position, with no influx of blood or liquid into the syringe.
8. Deviate the tip of the needle in the posterior direction, bypassing the lateral aspect of the mandibular condyle until it penetrates medially close to the back surface of the mandibular condyle. This space corresponds to the lower compartment. Next, slowly inject the viscosupplement (0.5 ml) and allow it to infiltrate.
9. For the upper compartment, open the mouth to its maximum width, and insert the needle at Point 1, with a 45-degrees slope in the posterior–anterior direction; advance it gently in the medial direction, until it is felt puncturing the joint capsule. The correct location of the needle tip must be checked by US examination and confirmed by negative pressure of the plunger. Once the correct position of the needle’s tip has been verified, slowly inject the viscosupplement (1 ml).

**Figure 7:**
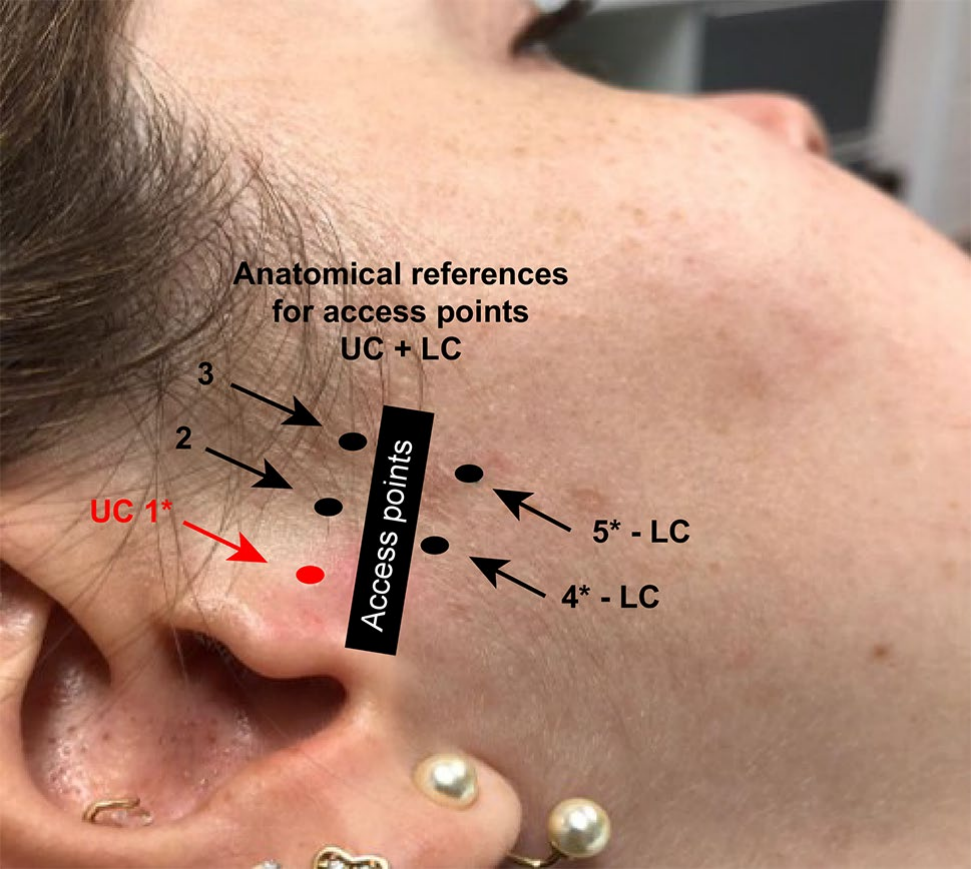
Reference points proposed by the evaluated protocol.

The marked points proposed by this protocol are represented in Fig. 7. They were applied in all stages of the present study, as described below.

### Ex vivo stage

The proposed protocol was initially tested in an ex vivo study with a sample of 10 heads of fresh frozen cadavers (n = 20 TMJs), made available for the study by the Department of Morphology of the Sciences Biological institute of the Federal University of Minas Gerais, respecting all ethical principles, and according to the approval of the institutional ethics committee mentioned above.

**Figure 8:**
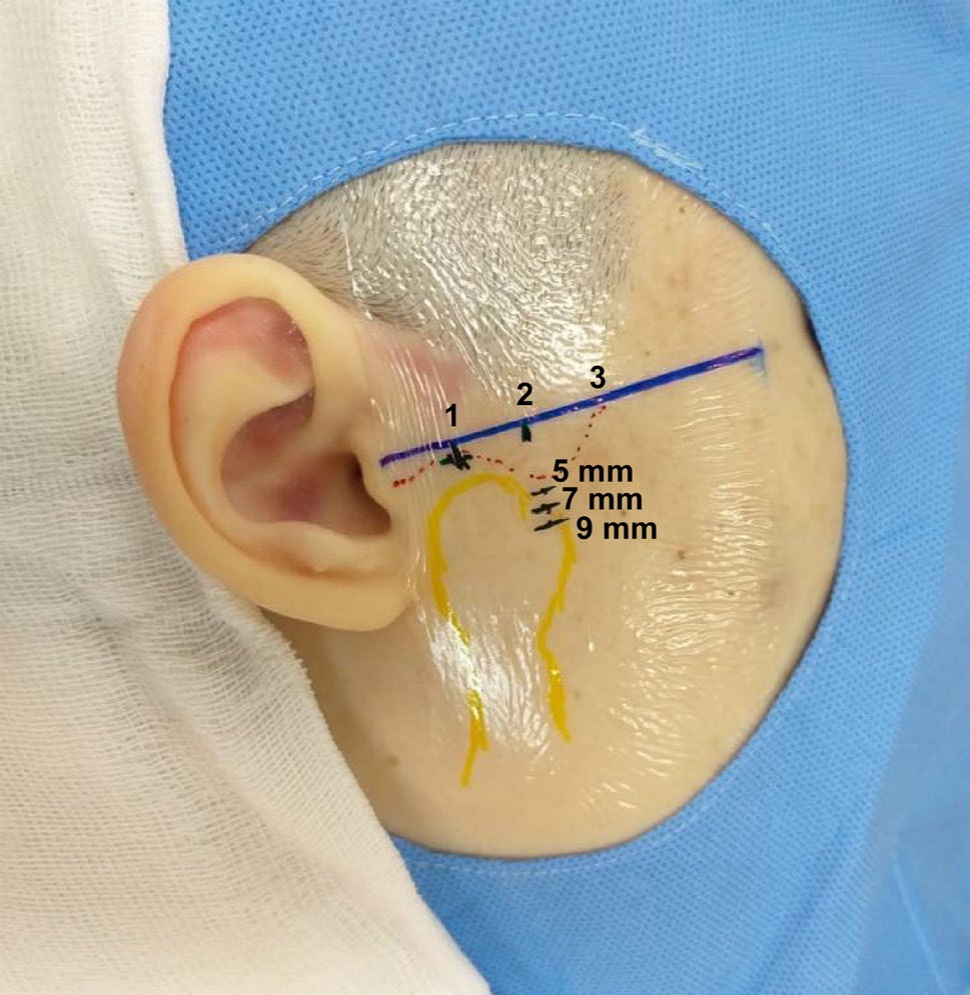
Reference points marked on a fresh frozen cadaver.

A pilot study was performed as a first stage of the ex vivo evaluation using three heads (A, B, C) that had their skin surfaces marked according to the proposed protocol bilaterally (n = 6 TMJs). To evaluate the effectiveness of the protocol for accessing the lower compartment, points below the line Tr-Ol line were made differently on each head (A: 5 mm; B: 7 mm; C: an additional point marked 9 mm below the Tr-Ol line) (Fig. 8).

After a colored was injected elastomer into the upper and lower compartments of each TMJ, according to proposed protocol, the TMJs were dissected to verify the location of the elastomer. The infiltrations in the head B, in which Points 4 was marked 7 mm below the Tr-Ol line, presented 100% accuracy. Heads A and B presented a 50% accuracy each.

In the second stage of the study, the access point to the lower compartment was standardized to a point 7 mm below the Tr-Ol line on the remaining 07 heads (14 TMJs). Silver-plated stainless-steel microspheres used in acupuncture (Complementar Agulhas para Acupuntura™—São João Del Rei, Minas Gerais—Brazil), were fixed on the skin, above all the marked points, using micropore adhesive tape. The heads were then assessed in CBCT scans (FOV = 70 × 70 mm; voxel = 0.2 mm; 120 kV; 5.0 mA; exposure time = 23 s—CB 500™—Gendex/Kavokerr. Moema, São Paulo, Brazil), to verify the correspondence of the marked points with the osseous components of the TMJ.

After CBCT exams, the microshperes were removed, and colored fluids were injected into the 14 TMJs, enabling their location in relation to be visualized in relation to surrounding tissues. The blue fluid was directed towards the upper compartment, while the redfluid was directed towards the lower compartment. After the injections, the TMJs of the fresh frozen cadavers were dissected to verify the accurate implementation of the proposed protocol.

### In vivo evaluation

The sample was composed of 10 individuals (n = 20 TMJs), with complaints of limitations of mouth opening and lateral movement, accentuated pain during joint movement, crackling or joint noise, with a clinical/imaging diagnosis of joint degeneration, adhesion, or chronic displacement of the articular disc related to a reduced volume or quality reduction of the synovial liquid. These patients attended at the post-graduate course of Orofacial Pain and Temporomandibular Dysfunction (Mater Dei Hospital/Neon Cursos—Belo Horizonte, Brazil), and VS was recommended. All the patients agreed to be included in the study by means of a written Free and Informed Consent Form. The patients who refused to participate in the study received normal care.

Before undergoing VS, the patients were examined by US to assess the anatomical aspects of the joints, to measure the thickness of the skin and subcutaneous tissues, and to map the vascular structures in the TMJ region. The thickness of the lateral joint space was measured as the distance between the lateral surface of the mandibular condyle and the lateral boundary of the joint capsule^31^. At the end of each procedure, a new US exam was conducted to perform a final measurement of the same region. One of the researchers, a dentomaxillofacial radiologist with 17 years of professional experience, evaluated all TMJs by means of US imaging (7–10 MHz linear transducer—Vscan™—GE Medical Systems—Milwaukee, WI, USA).

VS were then performed using HA (Osteonil™—TRB Chemedica Ag; Munich, Germany), in the upper and lower compartments of the TMJs, according to the proposed protocol. All of the infiltrations were performed by one of the researchers, a specialist with 34 years of experience in TMD and orofacial pain, with real-time verification of the needle tip locations by mean of US.

## Supplementary Information

Supplementary Video 1.

## Data Availability

The authors do not wish to share their raw data. The data cannot be published to avoid dual publication because they will be partially used in another study that is still being performed. Data are, however, available from the corresponding author upon reasonable request.
